# Multiregion WES of metastatic pancreatic neuroendocrine tumors revealed heterogeneity in genomic alterations, immune microenvironment and evolutionary patterns

**DOI:** 10.1186/s12964-024-01545-6

**Published:** 2024-03-06

**Authors:** Yu Jiang, Yi-han Dong, Shi-wei Zhao, Dong-yu Liu, Ji-yang Zhang, Xiao-ya Xu, Hao Chen, Hao Chen, Jia-bin Jin

**Affiliations:** 1 Department of General Surgery, Pancreatic Disease Center, Ruijin Hospital Affiliated to Shanghai Jiao Tong University School of Medicine, 197 Ruijn 2nd Road, Shanghai, 200025 People’s Republic of China; 2 Department of Pathology, Ruijin Hospital Affiliated to Shanghai Jiao Tong University School of Medicine, Shanghai, 200025 People’s Republic of China; 3 Department of Clinical and Translational Medicine, 3D Medicines Inc., Shanghai, 201114 People’s Republic of China; 4 Bioinformatics Department, JMDNA Inc., Building 23, 500 Furonghua Road, Shanghai, 201203 People’s Republic of China

**Keywords:** Pancreatic neuroendocrine tumors, Multiregion sampling whole-exome sequencing, *MEN1*/*DAXX*

## Abstract

Pancreatic neuroendocrine tumors (PanNETs), though uncommon, have a high likelihood of spreading to other body parts. Previously, the genetic diversity and evolutionary patterns in metastatic PanNETs were not well understood. To investigate this, we performed multiregion sampling whole-exome sequencing (MRS-WES) on samples from 10 patients who had not received prior treatment for metastatic PanNETs. This included 29 primary tumor samples, 31 lymph node metastases, and 15 liver metastases. We used the MSK-MET dataset for survival analysis and validation of our findings. Our research indicates that mutations in the *MEN1*/*DAXX* genes might trigger the early stages of PanNET development. We categorized the patients based on the presence (*MEN1*/*DAXX*^mut^, *n* = 7) or absence (*MEN1*/*DAXX*^wild^, *n* = 3) of these mutations. Notable differences were observed between the two groups in terms of genetic alterations and clinically relevant mutations, confirmed using the MSK-MET dataset. Notably, patients with mutations in *MEN1*/*DAXX*/*ATRX* genes had a significantly longer median overall survival compared to those without these mutations (median not reached vs. 43.63 months, *p* = 0.047). Multiplex immunohistochemistry (mIHC) analysis showed a more prominent immunosuppressive environment in metastatic tumors, especially in patients with *MEN1*/*DAXX* mutations. These findings imply that *MEN1*/*DAXX* mutations lead PanNETs through a unique evolutionary path. The disease’s progression pattern indicates that PanNETs can spread early, even before clinical detection, highlighting the importance of identifying biomarkers related to metastasis to guide personalized treatment strategies.

**Supplementary Information:**

The online version contains supplementary material available at 10.1186/s12964-024-01545-6.

## Background

Pancreatic neuroendocrine tumors (PanNETs) account for 1–2% of all pancreatic tumors, ranking as the second most common pancreatic malignancy [1]. Alarmingly, 40% of PanNET patients receive their diagnosis with distant metastases already present [2]. In the past two decades, the treatment of PanNETs has transformed significantly with the introduction of chemotherapy, targeted therapies, and peptide receptor radionuclide therapy. Despite these advances, metastatic disease often resists treatment, resulting in a 5-year survival rate of approximately 50% [3]. A critical challenge contributing to this issue is the limited understanding of genome heterogeneity and evolutionary patterns in metastatic PanNETs.

Metastasis is the primary cause of cancer-related deaths in PanNET patients and is considered the result of a complex process known as the invasion-metastasis cascade. Unlike other cancers like colorectal [4–6], breast [7], and pancreatic cancer [8], where metastatic dissemination timing and trajectory are extensively studied, key aspects, such as genome heterogeneity, molecular mechanisms, and evolutionary patterns associated with metastatic spread, are largely unexplored in PanNET metastasis, despite their clinical significance. Although several studies have characterized somatic mutations in PanNETs, few have examined the relationship between primary and metastatic lesions within individual PanNETs [9–12]. A challenge in metastasis research is the limited availability of matched primary tumor and metastasis pairs. However, with surgical resection even for metastatic PanNETs, applying multiregion sampling whole-exome sequencing (MRS-WES) holds promise in revealing genome heterogeneity and evolutionary patterns in metastatic PanNETs [4, 5].

Somatic mutations frequently occur in PanNETs, affecting genes linked to four key pathways: telomere maintenance (*DAXX* and *ATRX*), chromatin remodeling (*MEN1* and *SETD2*), DNA damage repair (*CHEK2*, *BRCA2*, and *MUTYH*), and mTOR signaling activation (*TSC2* and *PTEN*) [11, 12]. *DAXX* and *ATRX* genes encode chromatin remodeling proteins primarily facilitating the deposition of H3.3 histone variants in the telomere region [13]. Notably, one-third of PanNETs have inactivating mutations in *DAXX* or *ATRX* genes, resulting in a loss of nuclear expression of these proteins. These proteins act as independent prognostic biomarkers associated with shorter recurrence-free survival, even in cases of smaller tumors (≤ 2.0 cm) in patients with non-functioning PanNETs (NF-PanNETs) [14–16]. As a result, PanNETs with *DAXX*/*ATRX* mutations demonstrate an increased potential for metastasis. However, disparities between *DAXX*/*ATRX* mutated and wild-type metastatic PanNETs remain largely unexplored, considering factors such as genome heterogeneity, the tumor microenvironment, evolutionary patterns, and clinical relevance.

In summary, this study utilizes spatial genomic sampling of multiple tumors from ten treatment-naive patients to investigate genome heterogeneity, underlying molecular mechanisms, and evolutionary patterns in *MEN1*/*DAXX*-Mutated/Wild Type metastatic PanNETs. These findings provide valuable insights into tumor metastasis and offer potential implications for therapeutic strategies.

## Methods

### Sample collection

We gathered 113 FFPE samples from 12 consecutive patients diagnosed with metastatic PanNETs at our hospital between 2019 and 2020 (Table S1). Inclusion criteria for this study were as follows: (1) Both primary tumor (PT) and metastatic samples were treatment-naive; (2) each patient had matched white blood cells at the initial diagnosis; and (3) each patient had matched PT and metastatic samples. The follow-up for this patient cohort concluded on March 23, 2023. Ethics and scientific committees of our hospital approved this project, and all enrolled patients provided informed consent. To evaluate intratumor heterogeneity, we sampled multiple regions (*n* = 2–5) from the PT, with each section at least 0.5 cm apart from the others, depending on tumor size. Pathology results were independently verified by two experienced GI pathologists (WT, DYH) to ensure specimen quality. All specimens (*n* = 113) underwent whole-exome sequencing (WES). Paired PT and liver metastasis samples from each patient (*n* = 21) underwent multiplex immunohistochemistry (mIHC) to assess the tumor immune microenvironment.

### Sample processing and DNA extraction

The tumor content of formalin-fixed paraffin-embedded (FFPE) sample sections was estimated using hematoxylin and eosin (H&E) staining. Sections with a tumor content exceeding 20% were eligible for DNA extraction. FFPE sections were deparaffinized in a 1.5-microcentrifuge tube using mineral oil. They were then combined with lysis buffer and proteinase K and incubated at 56℃ overnight. Subsequently, the lysate underwent 80℃ for 4 h to reverse formaldehyde crosslinks. Genomic DNA was extracted from the lysate using the ReliaPrep™ FFPE gDNA Miniprep System (Promega) and quantified using the Qubit™ dsDNA HS Assay Kit (Thermo Fisher Scientific).

### Whole-exome sequencing

The extracted DNA (30–200 ng) underwent fragmentation into 250 bp fragments using an S220 focused-ultrasonicator (Covaris). Library preparation followed the KAPA Hyper Prep Kit (KAPA Biosystems) manufacturer’s protocol. The library’s concentration and size distribution were assessed using a Qubit 3.0 fluorometer (Thermo Fisher Scientific) and a LabChip GX Touch HT Analyzer (PerkinElmer), respectively. Four indexed DNA libraries (500 ng each) were pooled and mixed with Human Cot-1 DNA and xGen Universal Blockers-TS Mix, followed by drying down in a SpeedVac system. The pooled libraries were incubated at 95℃ for 10 min with the Hybridization Master Mix. Subsequently, 4 mL of the xGen Exome Research Panel v1.0 (IDT) was added, and the mixture was incubated at 65℃ overnight. Target capture followed the manufacturer’s instructions. The concentration and fragment size distribution of the final pooled library were determined as previously described. The final libraries were loaded onto a NovaSeq 6000 platform (Illumina) for 100 bp paired-end sequencing with an average sequencing depth of 200x.

### Data processing and variant calling

The raw data produced by the Illumina sequencer underwent alignment to the human reference genome, hg19, using the Burrows-Wheeler Aligner (v0.7.12) [17]. Subsequently, PCR duplicates were eliminated utilizing Picard (v1.130), and quality metrics were collected through Samtools (v1.1.19). Variant calling employed a custom R package, utilizing a binomial test-based variant detection model and the official BED file of the IDT WES panel. Local realignment was performed for accurate indel detection. Retained were SNVs with a depth of ≥ 30, supported by at least ≥ 4 reads, and with an allele frequency of > 3%, while indels larger than 40 bps were filtered out. Following the removal of potential FFPE artifacts, SNVs and indels were annotated using ANNOVAR with data from dbSNP (v138), 1000Genome, and ESP6500. Only SNVs and indels with a population prevalence of < 1% were included in subsequent analyses. Further details on variant calling and bias filtering from FFPE samples were previously described [18]. 

### Genome characterization

Sequenza [19] and FACETS [20] were employed to evaluate the ploidy and purity of the tumor samples. Samples with a purity of less than 30% were excluded. Intratumor heterogeneity (ITH) was assessed using the mutant-allele tumor heterogeneity (MATH) score. MSI was detected using a Python package developed in-house. Tumor mutation burden (TMB) was calculated by counting filtered mutations classified as missense, nonsense, nonstop, frameshift indel, in-frame indel, and splice site mutations. Tumor neoantigen burden (TNB) was calculated using NetMHCpan [21]. FACETS was used to detect allele-specific copy number variations, whole-genome duplications (WGD), and homologous recombination deficiency (HRD). The cellular prevalence of mutations (CCFs) and the clonality of mutations were estimated following established methodologies [5].

Mutation signatures were analyzed using maftools [22] and deconstructSigs [23]. De novo mutation signatures were constructed through non-negative matrix factorization (NMF) applied to all filtered mutations. The number of signatures was optimized based on cophenetic correlation. The three de novo signatures were deconvoluted to COSMIC v2 and v3 mutational signatures using cosine similarity to infer their biological significance.

### Multiplex immunohistochemistry (mIHC)

Multiplex immunohistochemistry (mIHC) was conducted using the Akoya OPAL Polaris 7-Color Automation IHC kit (NEL871001KT). Formalin-fixed, paraffin-embedded (FFPE) tissue slides underwent deparaffinization in a BOND RX system (Leica Biosystems). Subsequently, the slides were sequentially incubated with primary antibodies targeting specific markers, including CD163 (Abcam, ab182422, 1:500), CD68 (Abcam, ab213363, 1:1000), PD-1 (CST, D4W2J, 86163S, 1:200), PD-L1 (CST, E1L3N, 13684S, 1:400), CD3 (Dako, A0452), CD4 (Abcam, ab133616, 1:100), CD8 (Abcam, ab178089, 1:100), CD56 (Abcam, ab75813, 1:100), CD20 (Dako, L26, IR604), FOXP3 (Abcam, ab20034, 1:100), and pan-CK (Abcam, ab7753, 1:100) or S100 (Abcam, ab52642, 1:200) from Akoya Biosciences. This was followed by incubation with secondary antibodies and corresponding reactive Opal fluorophores. DAPI was used for nuclei counterstaining. Negative controls, consisting of tissue slides undergoing primary and secondary antibody binding but not exposed to fluorophores, were included to assess autofluorescence.

The multiplex-stained slides were scanned using a Vectra Polaris Quantitative Pathology Imaging System (Akoya Biosciences) at 20 nm wavelength intervals ranging from 440 to 780 nm, with a fixed exposure time and an absolute magnification of ×200. Subsequently, all scans for each slide were superimposed to generate a single composite image. Multilayer images were imported into inForm v.2.4.8 (Akoya Biosciences) and AP-TIME v.0.3.5 (3DMed) for quantitative image analysis. Tumor parenchyma and stromal regions were differentiated by Pan-CK staining. The quantities of various cell populations were reported as the number of stained cells per square millimeter and as the percentage of positively stained cells among all nucleated cells.

### Evolutionary analysis of tumors

REVOLVER [24] elucidated the trajectories of recurrent evolution using multi-region sequencing data from 10 patients. These patients were categorized into Mut and WT groups based on *MEN1*/*DAXX* mutations. Subsequently, the top 20 frequently mutated genes from each group were selected for further analysis.

To construct a matrix representing all filtered mutations in multi-region samples for each patient, we assigned a value of 1 to mutations and 0 to wild-type status. This matrix was then utilized with the R packages Phangorn [25] and MesKit [26] to build the phylogenetic tree. The neighbor-joining (NJ) and maximum parsimony (MP) algorithms were employed for this purpose, and bootstrapping with 100 replicates was performed to estimate the confidence of the phylogenetic tree.

### Identification of metastasis-selected events

For the identification of metastasis-selected events, primary and metastatic samples were paired (33 pairs in the Mut group and 13 pairs in the WT group). Metastasis-selected events were defined as variants exclusively present in metastasis samples or variants subclonal in primary samples but becoming clonal in metastasis samples. Only variants present in ≥ 60% of patients in either the Mut or WT group were considered.

### Timing of dissemination

The SCIMET [5] tool was employed to determine the timing of dissemination. It estimated the mutation rate per cell division (u) and the primary tumor size at the time of dissemination using posterior probabilities based on CCFs of SNVs in primary-metastasis pairs. Early dissemination occurred before reaching a cell count of *N* = 10^8^ cells, while late dissemination was characterized by reaching Nd ≥ 10^8^ cells.

### Analysis on the MSK-MET dataset

Primary or metastatic samples from a total of 25,000 patients in the MSK-MET (Memorial Sloan Kettering - Metastatic Events and Tropisms) dataset, including 189 patients with metastatic Pancreatic Neuroendocrine Tumors, were selected. Clinical data, mutations, copy number variations (CNV), and microsatellite instability (MSI) results for these samples were obtained from cBioPortal. Tumors exhibiting at least one mutation or CNV event were included for further analysis.

### Quantification and statistical analysis

All statistical analyses were conducted using Python (version 3.8). The Mann-Whitney U test was employed for comparing independent continuous data. Fisher’s exact test was used for comparing proportions between groups. Survival analyses were performed using Kaplan-Meier curves, and comparisons were made using the log-rank test. To address multiple hypothesis testing, the Benjamini-Hochberg method was applied. All hypothesis tests were two-sided, and statistical significance was determined at a *p*-value or false discovery rate (FDR) < 0.05.

## Results

### Overview of the PanNET cohort and the study design

**Fig. 1:**
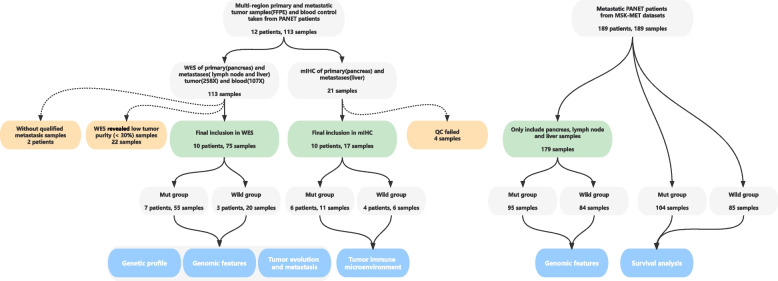
The flowchart of study design. Using Single-Nucleotide Variant (SNV) profiles derived from WES data, we categorized patients into two groups: those with mutated *MEN1*/*DAXX* (*MEN1*/*DAXX*^mut^, *n* = 7) and those with wild-type *MEN1*/*DAXX* (*MEN1*/*DAXX*^wild^, *n* = 3). Subsequently, we conducted analyses to explore differences between these two groups in the following aspects: (1) heterogeneity in genomic alterations, (2) characteristics of the immune microenvironment, and (3) evolutionary patterns

We enrolled 12 treatment-naive patients with metastatic PanNET for this study (Table S1), and all provided matched primary-metastasis pairs. Among these patients, 91.7% (11/12) had synchronous metastases, with only one exhibiting metachronous metastasis, where the metastatic tumors were identified 12.1 to 18.8 months after the initial surgery. Multiregion sampling (MRS) was performed on the primary, lymph node, and liver metastatic tumors in these individuals (Fig. 1). Depending on tumor size, 2 to 5 specimens were harvested from each primary tumor. Subsequently, WES was conducted on a total of 113 specimens obtained from MRS. Two patients (P8 and P9) were excluded from subsequent genomic and evolutionary analyses due to the absence of matched primary-metastasis pairs after quality control. Among the remaining samples, 75 passed quality control, comprising 29 specimens from primary tumors, 31 from lymph node metastases, and 15 from liver metastases (Fig. 1, Table S2). Additionally, mIHC was conducted on 16 specimens that met quality control criteria to investigate the immune microenvironment. For validation, the MSK-MET (Memorial Sloan Kettering-Metastatic Events and Tropisms) public database [27] was utilized, including 189 metastatic PanNET patients (Table S3).

### Genetic profiling of PanNET samples and spatial heterogeneity

**Fig. 2:**
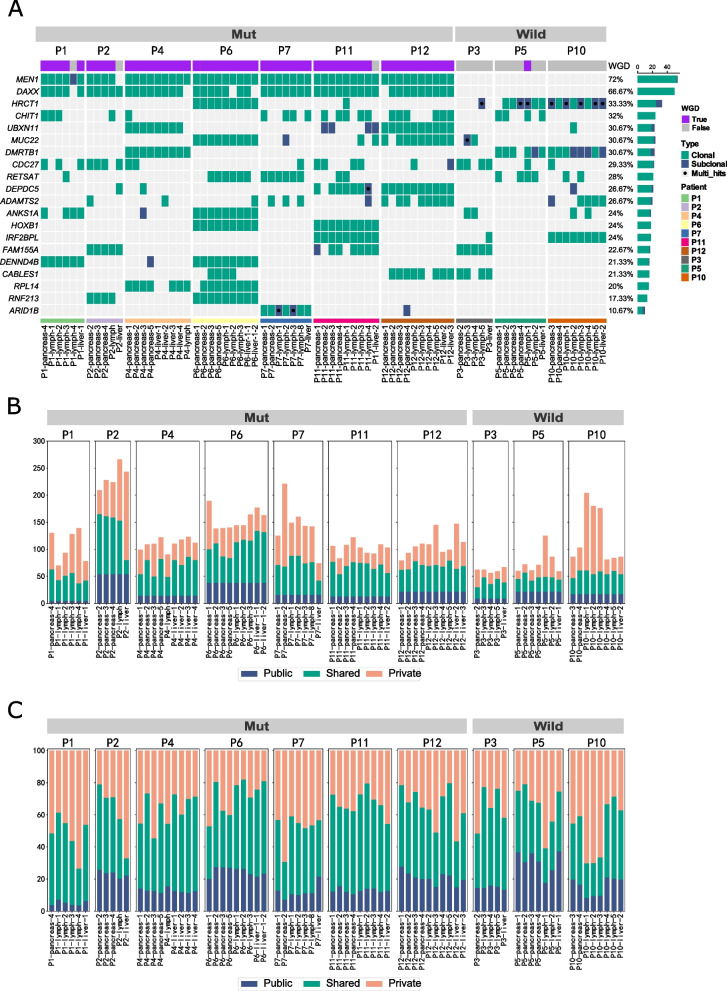
Genomic profile of PanNET cohort. **A** Genetic profiles: the genetic profiles of 75 primary and metastatic tumor samples from 10 patients are depicted in this panel. Each line represents mutations of a single gene across all samples. The top 20 frequently mutated genes are listed in descending order on the leftmost side. The mutation frequency of each gene is indicated to the right, followed by the numbers of clonal (in green) and subclonal (in blue) mutations. Each column represents mutations in a single sample, with the sample ID and patient’s information (indicated by the colorbar) listed at the bottom. **B** Distribution of Nonsynonymous somatic mutations: the distribution of nonsynonymous somatic mutations is categorized into private, shared, and public mutations across all samples. Private mutations, occurring only once, are colored in apricot. Shared mutations, found in at least two samples within the same patient, are depicted in green. Public mutations, shared by all samples within one patient, are shown in navy blue. The vertical axis represents the mutation counts, while the horizontal axis displays the sample IDs (at the bottom) grouped by patient (at the top). **C** Proportions of private, shared, and public nonsynonymous somatic mutations: this chart illustrates the proportions of private, shared, and public nonsynonymous somatic mutations in all samples

The MRS-WES analysis revealed a total of 9067 SNV/Indel, distributed among primary tumors (3504), lymph nodes (3790), and liver metastases (1773). Of these, 6874 were identified as clonal variants (75.81%), while 2193 were categorized as subclonal variants (24.19%). The genes with the highest mutation frequencies in this cohort, listed in descending order, were *MEN1* (72%), *DAXX* (66.67%), *HRCT1* (33.33%), *CHIT1* (32%), and *UBXN11* (30.67%) (Fig. 2A). Spatial intra-tumor heterogeneity (ITH) was evident in all 10 PanNET samples, with an average of 82.6% of somatic variants displaying spatial heterogeneity (Range: 62.7–96.4%), indicating a parallel evolution model. Across the ten patients, only 17.12% of the variants (Public) were present in all patient-provided samples, 42.84% of the variants (Shared) were observed in more than one sample, and 40.05% of the variants (Private) were exclusive to a single sample from the patient (Fig. 2B).

Figure 2A illustrates that *MEN1* and *DAXX* mutations occurred at a frequency more than double that of any other gene. The majority of variants observed in these two genes were clonal (Clonal: 109 out of 111, 98.20%), whereas other genes were predominantly associated with subclonal variants (Clonal: 6765 out of 8956, 75.54%). Variants of *MEN1* and *DAXX* were found in nearly all samples from the carrier population, demonstrating a robust presence not observed in variants of other genes (Mean variant prevalence: 94.29% in *MEN1*/*DAXX* vs. 25.11% in others). Additionally, the mean Cancer Cell Fractions (CCF) of *MEN1*/*DAXX* variants were significantly higher when compared to other genes (Mean CCF: 96.32% in *MEN1*/*DAXX* vs. 80.27% in others). Remarkably, variants of *MEN1* and *DAXX* co-occurred in a large majority of cases (Co-occurrence: 89.09%). These findings suggest that *MEN1* and *DAXX* may jointly initiate oncogenesis at the earliest stage of PanNET development. Consequently, PanNETs with *MEN1*/*DAXX*^wild^ mutations may potentially follow an alternative cancer development pathway. Motivated by this possibility, we divided the 10 patients into two groups (*MEN1*/*DAXX*^mut^ and *MEN1*/*DAXX*^wild^) and explored differences between these groups in relation to cancer formation. Patients carrying the *MEN1*/*DAXX* mutation exhibited slightly higher spatial heterogeneity with an average of 83.8% (range: 72.2-96.4%), in comparison to wild-type carriers (79.3%, range [62.7-91.7%], *p* = 0.028).

### Genomic discrepancies between *MEN1/DAXX*^mut^ and *MEN1/DAXX*^wild^ groups

**Fig. 3:**
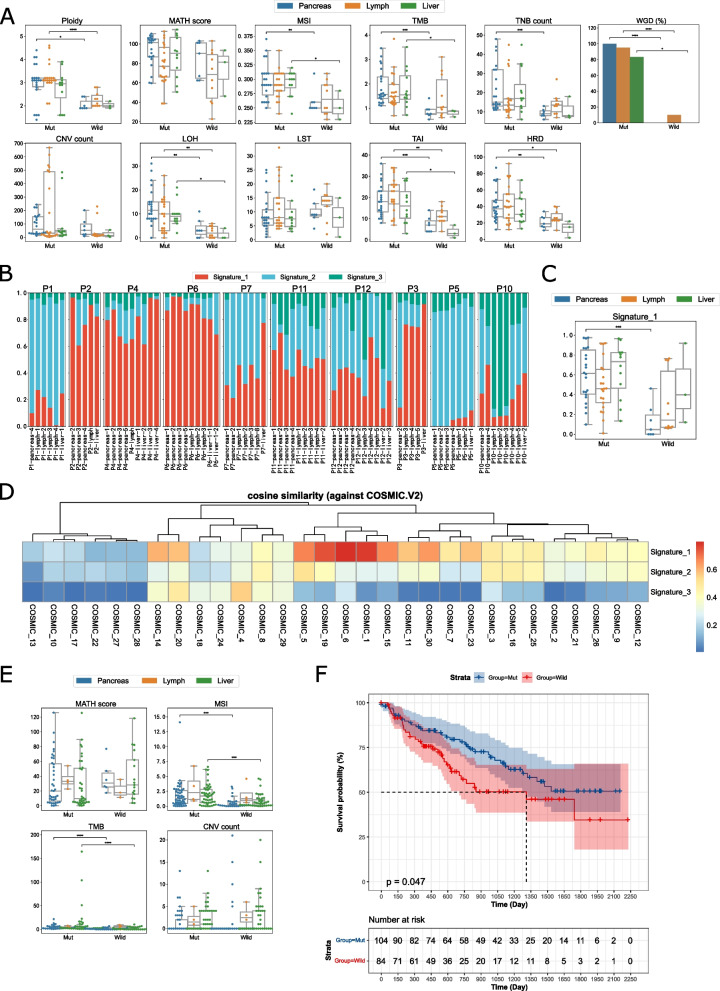
Genomic difference between *MEN1*/*DAXX*^mut^ and *MEN1*/*DAXX*^wild^ group. **A** This chart displays the distribution of various genomic features—ploidy, MATH score, MSI, TMB, TNB, CNV counts, LOH, LST, TAI, HRD, and WGD—in primary tumors (blue, primary), lymph node metastases (orange, metastatic), and liver metastases (green, metastatic) within the *MEN1*/*DAXX*mut and *MEN1*/*DAXX*^wild^ groups. The vertical axes represent the values of the respective genomic features, while the horizontal axes distinguish between the *MEN1*/*DAXX*^mut^ and *MEN1*/*DAXX*^wild^ groups. Each data point corresponds to a single sample. **B** This chart presents the composition of de novo mutational signatures across all 75 samples. The vertical axis represents the percentage of each de novo mutational signature, with De novo Signatures 1, 2, and 3 colored in cinnabar, turquoise, and green, respectively. Sample IDs are listed at the bottom, grouped by patient IDs at the top. **C** The distribution of de novo Signature 1 composition in primary tumors (blue, primary), lymph node metastases (orange, metastatic), and liver metastases (green, metastatic) within the *MEN1*/*DAXX*^mut^ and *MEN1*/*DAXX*^wild^ groups is illustrated in this chart. The vertical axis represents the composition of de novo Signature 1, while the horizontal axis distinguishes between the *MEN1*/*DAXX*^mut^ and *MEN1*/*DAXX*^wild^ groups. Each data point represents a single sample. **D** Cosine similarity between de novo mutational signatures and COSMIC Signature V2 is visualized in this chart. The vertical axis represents the de novo signatures, while the horizontal axis represents COSMIC V2 signatures. The color of each tile in the figure indicates the cosine similarity between the horizontal COSMIC V2 signature and the vertical de novo signature, ranging from blue (lowest similarity) to cinnabar (highest similarity). **E** The distributions of MATH score, MSI, TMB, and CNV counts between *MEN1*/*DAXX*/*ATRX*^mut^ and *MEN1*/*DAXX*/*ATRX*^wild^ groups in the MSK-MET dataset are presented. Each data point represents a single sample, with blue dots representing pancreas primary samples, orange dots representing lymph samples, and green dots representing liver samples. The horizontal axis distinguishes between the *MEN1*/*DAXX*/*ATRX*^mut^ and *MEN1*/*DAXX*/*ATRX*^wild^ groups, while the vertical axis represents the values of the respective genomic features. **F** Overall survival analysis using Kaplan-Meier for patients with or without *MEN1*/*DAXX*/*ATRX* mutations in the MSK-MET dataset is displayed. The analysis includes all patients (*n* = 188) with available follow-up information

We compared genomic features in two groups, categorized by primary, lymph node, and liver metastases (Fig. 3A, Table S2). Our analysis found that *MEN1*/*DAXX*^mut^ samples showed higher levels of tumor mutation burden (TMB: Primary 1.88 vs. 0.87, *p* < 0.001; Lymph node 1.68 vs. 1.46, *p* = 0.176; Metastatic 1.81 vs. 0.80, *p* = 0.018), tumor neoantigen burden (TNB: Primary 21.86 vs. 9.29, *p* < 0.001; Lymph node 17.89 vs. 13.9, *p* = 0.648; Metastatic 20.5 vs. 11, *p* = 0.219), and microsatellite instability (MSI: Primary 0.297 vs. 0.261, *p* = 0.007; Lymph node 0.291 vs. 0.268, *p* = 0.052; Metastatic 0.293 vs. 0.253, *p* = 0.049).

By using maftools [22], we identified three de novo mutation signatures from all samples. Although the proportions of these signatures varied significantly (Fig. 3B), the *MEN1*/*DAXX*^mut^ group, on average, had a higher signature 1 compared to the *MEN1*/*DAXX*^wild^ group (Signature 1: Primary 0.60 vs. 0.13, *p* < 0.001; Lymph node 0.49 vs. 0.31, *p* = 0.079; Metastatic 0.64 vs. 0.48, *p* = 0.536, Fig. 3C). As Signature 1 strongly correlates with COSMIC Signature 6 (COSMIC signatures V2), indicating dMMR (Fig. 3D, Figure S1), it can be inferred that *MEN1*/*DAXX*^mut^ samples were enriched for variants caused by dMMR.

The *MEN1*/*DAXX*^mut^ group showed a notable inclination towards Homologous Recombination Deficiency (HRD). Loss Of Heterozygosity (LOH) and Telomeric Allelic Imbalance (TAI) were significantly higher in this group compared to *MEN1*/*DAXX*^wild^ values (*MEN1*/*DAXX*^mut^ vs. *MEN1*/*DAXX*^wild^, LOH: 11.20 vs. 2.80, *p* < 0.001; TAI: 19.35 vs. 8.80, *p* < 0.001). HRD scores for primary pancreatic and lymph node metastasis in the *MEN1*/*DAXX*^mut^ group were statistically elevated compared to those in the *MEN1*/*DAXX*^wild^ group (*MEN1*/*DAXX*^mut^ vs. *MEN1*/*DAXX*^wild^, Primary: 41.32 vs. 21.14, *p* = 0.006; Lymph node: 43.67 vs. 26.7, *p* = 0.049, Fig. 3A). Additionally, the *MEN1*/*DAXX*^mut^ group exhibited higher ploidy levels than the *MEN1*/*DAXX*^wild^ group (Primary: 3.00 vs. 2.06, *p* = 0.013; Lymph node: 3.23 vs. 2.28, *p* < 0.001; Metastatic: 2.77 vs. 2.03, *p* = 0.217) and a higher level of whole genome duplication (*MEN1*/*DAXX*^mut^ vs. *MEN1*/*DAXX*^wild^, WGD: 94.55% vs. 5.00%, *p* < 0.001, Fig. 3A). This could likely result from *MEN1*/*DAXX* dysfunction leading to defects in centromere cohesion due to ectopic CENP-A deposition, subsequently resulting in selective chromosome loss followed by whole genome duplication [13].

### Validation and survival analysis in the MSK-MET cohort

We analyzed the MSK-MET cohort, comprising 189 patients with metastatic pancreatic neuroendocrine tumors, to validate and perform survival analysis. Given the functional relevance of the *DAXX* and *ATRX* genes, we conducted validations comparing patients with *MEN1*/*DAXX*/*ATRX* mutations to those without (Table S3). Similar trends in genome features were observed between patients with and without *MEN1*/*DAXX*/*ATRX* mutations in this dataset. Notably, patients harboring *MEN1*/*DAXX*/*ATRX* mutations exhibited higher MSI and TMB values compared to those without these mutations, with no significant differences in the MATH score and CNV count (MSI: MUT 1.93 vs. WT 0.81, *p* < 0.001; TMB: MUT 7.66 vs. WT 2.17, *p* < 0.001, Fig. 3C). Subsequently, survival analysis was performed on both groups using the MSK-MET dataset (Fig. 3D, Figure S2). Interestingly, patients carrying *MEN1*/*DAXX*/*ATRX* mutations demonstrated a significantly higher overall survival rate compared to patients with the wild-type (median OS: not reached vs. 43.63 months, Log-rank test, *p* = 0.047).

### Clinically actionable somatic alterations

**Fig. 4:**
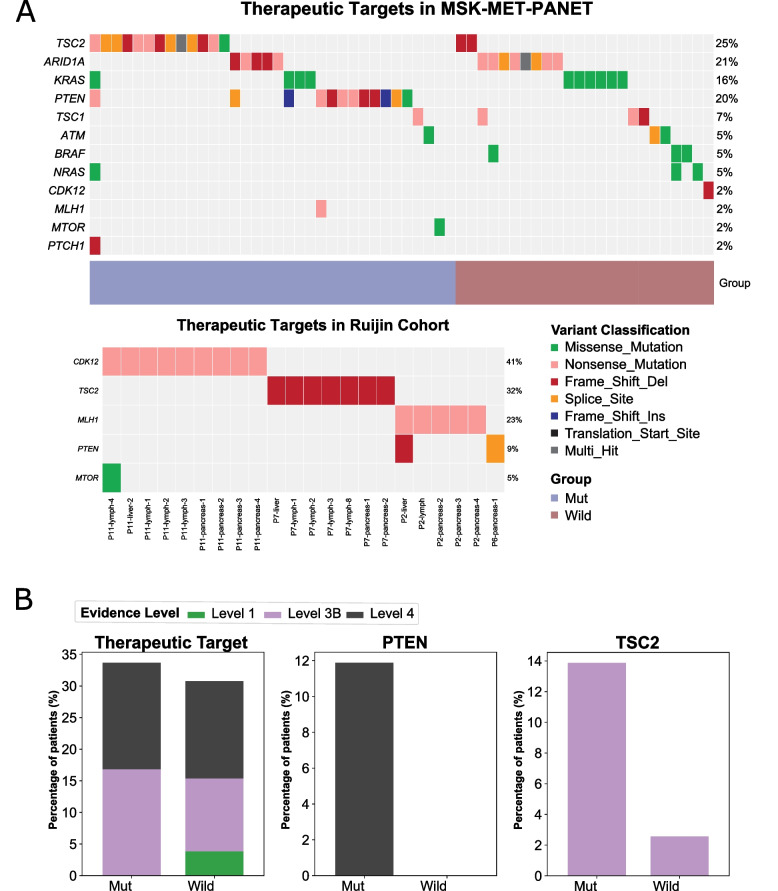
Clinically actionable somatic alterations observed in our and the MSK-MET cohort. **A** Utilize the OncoKB dataset to annotate clinically actionable somatic alterations within both the MSK-MET dataset (above) and our cohort (below), and subsequently present the findings in an oncoplot. **B** Investigate the variation in the proportion of clinically actionable somatic alterations between patients with and without *MEN1*/*DAXX*/*ATRX* mutations in the MSK-MET dataset. No significant difference is identified in the overall ratio (left graph, *p* = 0.158). However, individuals with *MEN1*/*DAXX*/*ATRX* mutations manifest significantly elevated mutation rates in *PTEN* (*p* = 0.0004) and *TSC2* (*p* = 0.0036) compared to those lacking these mutations

In our cohort, 4 out of 75 specimens (24%) from 4 patients harbored somatic aberrations recognized as potential targets for currently available or under-development treatment agents (Fig. 4A). It’s noteworthy that all these patients carried *MEN1*/*DAXX* mutations. For instance, patient P11 had a trunk mutation in *CDK12*, suggesting a likely response to various DNA damaging agents, such as etoposide. Additionally, *CDK12* inactivation renders tumors susceptible to immune checkpoint inhibitors, as observed in cases of mCRPC. Another patient, P2, possessed a trunk mutation in *MLH1*, resulting in the highest TMB in our cohort, potentially making them responsive to ICBs. All four patients had somatic mutations in *TSC2*, *PTEN*, and *MTOR*, all playing roles in the PI3K/AKT/MTOR pathway. Consequently, these patients could potentially benefit from treatment with everolimus. It’s worth noting that P7, with a trunk mutation in *TSC2*, received the mTOR inhibitor Everolimus after the last progression, associated with tumor regression.

In the MSK-MET cohort, 61 out of 189 patients (32.3%) harbored 70 actionable alterations (Fig. 4A, Table S4). Frequently observed alterations included *TSC2* (*n* = 17), *ARID1A* (*n* = 14), *PTEN* (*n* = 12), and *KRAS* (*n* = 10). When stratified by levels of evidence, the prevalence of targetable alterations was as follows: level 1 (1.6%), level 3B (15.9%), and level 4 (19.6%) (Table S2). These findings support the exploration of targeted therapies alone or in combination with immunotherapy or cytotoxic chemotherapy in selected PanNET populations. No differences in the proportions of patients with actionable mutations were observed between the two groups (Fig. 4B). However, some meaningful features still emerged. Three *MEN1*/*DAXX*/*ATRX* wild-type patients carried *BRAF p.V600E* mutations (Fig. 4B). Alterations in the PI3K/AKT/MTOR pathway were most frequent in the cohort (32 patients [16.9%]), with *TSC2* (11.5% vs. 0%, *p* < 0.001) and *PTEN* (14.4% vs. 2.4%, *p* = 0.004) mutations being more prevalent in the MUT group (Fig. 4B). Importantly, actionable alterations in the PI3K/AKT/MTOR pathway were more likely to coexist with *MEN1*/*DAXX*/*ATRX* mutations (26.9% vs. 5.9%, *p* < 0.001), aligning with our cohort observations. This suggests potential benefits from targeted therapy for patients carrying *MEN1*/*DAXX*/*ATRX* mutations, particularly focusing on the PI3K/AKT/MTOR pathway. Surprisingly, thirteen patients (13/189, 6.9%) exhibited high tumor mutation burdens (TMB-H, ≥ 10), indicating potential sensitivity to ICB therapy.

### Differences of immune microenvironments between MUT and WT groups

**Fig. 5:**
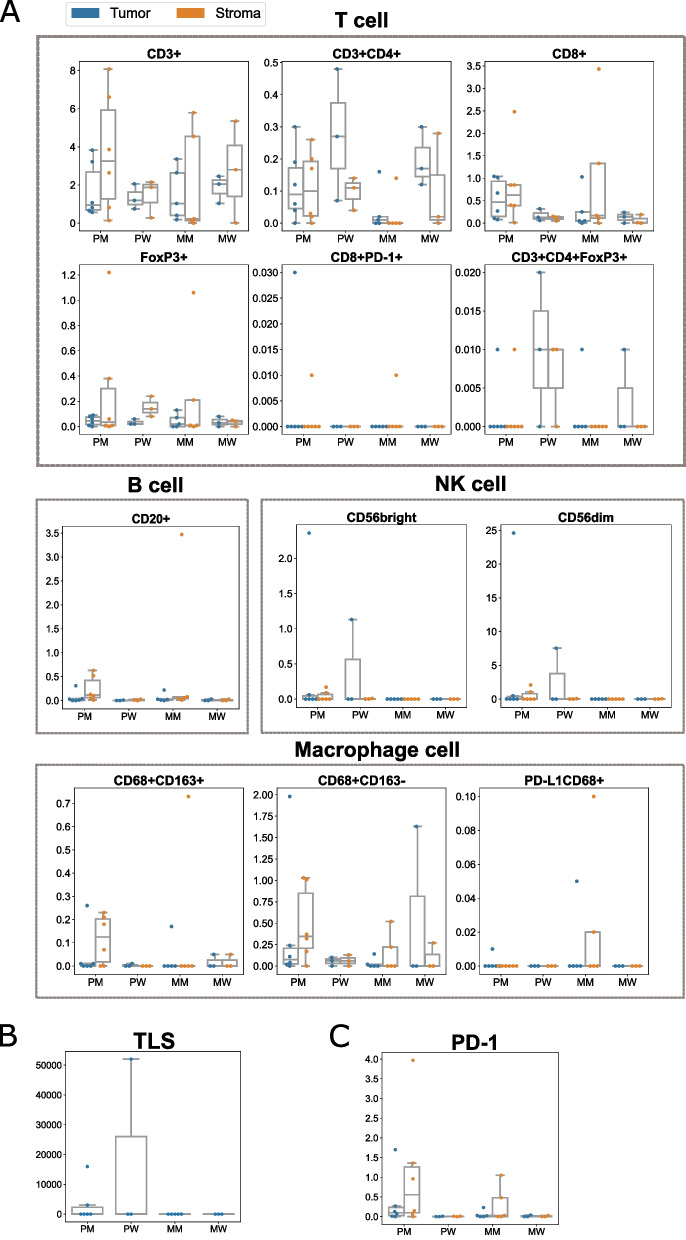
Difference of immune microenvironments between *MEN1*/*DAXX*^mut^ and *MEN1*/*DAXX*^wild^ groups. **A** The graphs depict the distributions of immune cell biomarker levels in the PM (Primary *MEN1*/*DAXX*^mut^), PW (Primary *MEN1*/*DAXX*^wild^), MM (Metastatic *MEN1*/*DAXX*^mut^), and MW (Metastatic *MEN1*/*DAXX*^wild^) groups. The vertical axes show biomarker levels, with orange bars indicating stromal levels and blue bars indicating tumor levels. Each dot on the graph represents one sample, and the name of each biomarker is provided at the top of the plots. **B** The graph illustrates the counts of tertiary lymphoid structures (TLS) in the PM, PW, MM, and MW groups. The vertical axes indicate TLS counts, and the horizontal axes categorize the groups. Each dot on the graph represents one sample. **C** The graphs present the distributions of PD-1 levels in the PM, PW, MM, and MW groups. The vertical axes represent PD-1 levels, while the horizontal axes denote the groups. Orange bars indicate PD-1 levels in the stroma, and blue bars represent PD-1 levels in the tumor. Each dot on the graph corresponds to one sample

We conducted a comprehensive exploration of the immune microenvironments in both *MEN1*/*DAXX*^mut^ and *MEN1*/*DAXX*^wild^ groups through multiplex immunohistochemistry (mIHC) analysis (Fig. 5, Figures S3-S8). Distinct patterns were observed in T cells with CD3 + and CD4 + between the two groups. In the *MEN1*/*DAXX*^mut^ group, these cells exhibited a decrease from the primary (PM) to the liver metastasis (MM), both within the tumor and stroma (*p* = 0.205 in tumor, *p* = 0.075 in stroma). Notably, the levels of CD68 + CD163-, indicative of M1 macrophages, were higher in the primary *MEN1*/*DAXX*^mut^ (PM) group compared to its metastatic (MM) counterpart (*p* = 0.280 in tumor, *p* = 0.332 in stroma). Conversely, no significant difference was observed in the *MEN1*/*DAXX*^wild^ group. CD20, a B cell marker, showed significant enrichment in the stroma of the primary *MEN1*/*DAXX*^mut^ (PM) group as opposed to the primary *MEN1*/*DAXX*^wild^ (PW) group (*p* = 0.036). On average, tertiary lymphoid structures (TLS) were more prevalent in primary locations than in metastatic ones (8879.89 μm²/mm² in primary, 0 in metastatic, *p*-value: 0.076). These results collectively suggest a more potent immunosuppressive microenvironment in metastatic tumors, particularly within the *MEN1*/*DAXX*^mut^ group.

### Tumor evolutions from primary to metastatic

**Fig. 6:**
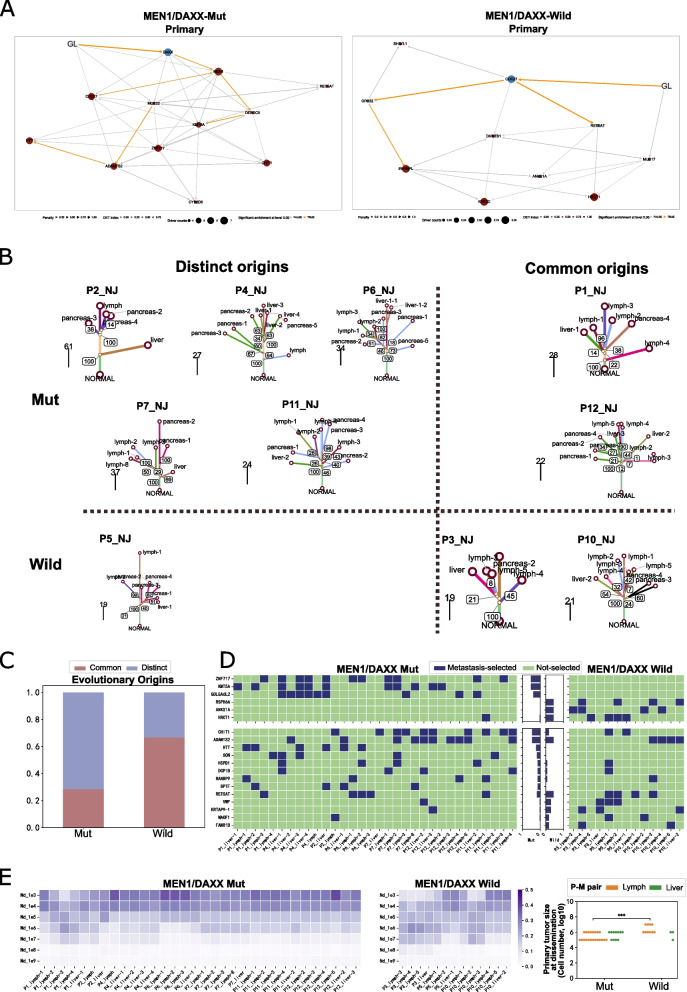
Difference of evolutionary trajectories between *MEN1*/*DAXX*^mut^ and *MEN1*/*DAXX*^wild^ groups. **A** Illustrating the evolutionary trajectories of the *MEN1*/*DAXX*^mut^ group (left) and *MEN1*/*DAXX*^wild^ group (right). “GL” denotes “Germline.” **B** Presenting the phylogeny reconstructed using the neighbor-joining method for the ten patients. Bootstrapping percentages from 100 repetitions are indicated at each node. **C** Depicting the proportions of evolutionary origins in both the *MEN1*/*DAXX*^mut^ and *MEN1*/*DAXX*^wild^ groups. The left axis represents the proportion of each origin, with blue columns indicating Distinct Origins and green columns representing common origins. **D** Highlighting metastasis-selected events in both the *MEN1*/*DAXX*^mut^ and *MEN1*/*DAXX*^wild^ groups. The left section displays metastasis-selected mutation events in the *MEN1*/*DAXX*^mut^ group, while the right section is for the *MEN1*/*DAXX*^wild^ group. The middle section indicates the proportion of metastasis-selected events in both groups. The upper section showcases metastasis-selected events with significantly different frequencies between the *MEN1*/*DAXX*^mut^ and *MEN1*/*DAXX*^wild^ groups, while the lower section displays metastasis-selected events without significantly different frequencies. Blue grids denote metastasis-selected mutations, while green grids indicate no metastasis-selected mutations. **E** Detailing mutation rates and the timing of dissemination across all patients. The left and middle sections display the SCIMET-estimated probability of metastasis timing for each primary-metastasis pair in the *MEN1*/*DAXX*^mut^ and *MEN1*/*DAXX*^wild^ groups, respectively. The color of each grid corresponds to the probability value. The right section provides a comparison of primary tumor sizes at the time of dissemination between the *MEN1*/*DAXX*mut and *MEN1*/*DAXX*^wild^ groups. The horizontal axis represents the *MEN1*/*DAXX*^mut^ and *MEN1*/*DAXX*^wild^ groups, while the vertical axis represents the primary tumor size at the time of dissemination. Each dot represents one primary-metastasis pair, with orange dots indicating pairs where metastasis occurred in lymph nodes and green dots indicating pairs where metastasis occurred in the liver

The evolutionary trajectories of *MEN1*/*DAXX*^mut^ and *MEN1*/*DAXX*^wild^ groups were delineated using the REVOLVER tool based on multi-region sequencing [24] (Fig. 6A). Distinct driving events and evolutionary paths characterized these two groups. *MEN1*/*DAXX*^mut^ group exhibited early mutations in *DAXX* and *MEN1*, followed by *CDC27* and *DEPDC5* mutations. In contrast, *MEN1*/*DAXX*wild group’s initial event was *CDC27* mutation. Metastasis-selected events, more prevalent in metastasis compared to primary tumor, were identified for both groups: *ZNF717, KMT5A*, and *GOLGA6L2* in *MEN1*/*DAXX*^mut^ metastasis, and *RSPH6A*, *ANKS1A*, and *HRCT1* in *MEN1*/*DAXX*^wild^ metastasis (Fig. 6D), suggesting divergent evolutionary routes in metastasis for each group.

Metastasis, a leading cause of cancer-related deaths, necessitates understanding precise evolutionary pathways for effective screening and surgical planning. The evolutionary tree revealed that 66.7% of liver metastases shared the same origin as the primary tumor (PT) or a subclone, differing from lymph node metastases. *MEN1*/*DAXX*^mut^ patients exhibited distinct origins (71.4%), while *MEN1*/*DAXX*^wild^ patients favored common origins (66.7%), indicating varied metastasis modes (Fig. 6C).

Paired primary tumors and metastases formed separate phylogenetic clades with high spatial intra-tumor heterogeneity (ITH), suggesting early metastatic dissemination. SCIMET model estimated early dissemination in all primary-metastasis pairs, with *MEN1*/*DAXX*^mut^ group disseminating significantly earlier than *MEN1*/*DAXX*^wild^ group in primary-lymph node pairs (*p* < 0.001, Fig. 5E). Metastatic process in PanNETs follows a parallel evolution model, determining invasion potential early.

P7 with metachronous metastases showed *MEN1*, *TSC2*, and *DAXX* mutations in all samples, indicating their crucial role in tumorigenesis. Evolutionary trees and dissemination timing revealed early dissemination even before clinical detection, emphasizing potential benefits of adjuvant treatment for early-stage patients with high metastatic risk.

## Discussions

While prior studies have predominantly focused on single lesions, offering limited insight into spatial heterogeneity and evolutionary patterns of PanNETs, our study employs MRS-WES, providing a pioneering and comprehensive exploration of these critical aspects [9–12, 28]. The results reveal a substantial intratumor heterogeneity (ITH) in PanNETs, with 82.6% of somatic variants displaying spatial heterogeneity on average. This heightened heterogeneity emphasizes the necessity of multi-site or repeated biopsies, crucial for informed therapeutic decision-making. Notably, *MEN1*/*DAXX*^mut^ and *MEN1*/*DAXX*^wild^ cases exhibit significant disparities in genome alterations and clinically actionable somatic mutations. The application of the SCIMET model and systems evolution analysis demonstrates that the majority of liver metastases originate directly from the primary site. Despite divergent metastatic pathways between *MEN1*/*DAXX*^mut^ and *MEN1*/*DAXX*^wild^ cases, PanNETs are generally categorized as early disseminated tumors, underscoring the intrinsic invasiveness and metastatic potential of specific tumors. Decoding the genomic diversity and evolutionary processes of PanNETs contributes not only to theoretical understanding but also establishes a translational foundation for identifying therapeutic targets and devising personalized treatment strategies.

Previous research, including our studies, has proposed that *MEN1* and *DAXX* mutations may serve as initiating events in PanNET development, potentially inducing telomere and DNA damage, thereby fostering tumorigenesis and progression [9, 13]. These mutations are pivotal drivers of PanNETs, often co-occurring, influencing tumor phenotype, immune microenvironment, treatment resistance, and prognosis. Our findings reveal that patients with *MEN1*/*DAXX* mutations exhibit higher TMB, TNB, and MSI scores. Three de novo mutational signatures identified by the NMF method, notably Signature 1 correlated with COSMIC Signature 6, associated with defective DNA mismatch repair, suggest that patients with *MEN1*/*DAXX* mutations may benefit from immune checkpoint blockade (ICB) treatments. Validation using the MSK-MET database corroborates these results. The observed median TMB aligns with a recent study on advanced PanNETs, categorizing them as ‘cold’ tumors in terms of their immune microenvironment. Converting the immune microenvironment from ‘cold’ to ‘hot’ is a crucial focus in PanNET immunotherapy. The use of the alkylating agent temozolomide (TMZ) has been associated with hypermutations, suggesting a potential strategy to modify the immune microenvironment of PanNETs. While TMZ is recommended in current treatment guidelines, its association with increased TMB raises interest in its potential synergy with ICB. Ongoing clinical trials are evaluating the efficacy of ICB in recurrent high-mutation gliomas and glioblastoma, and studies in mCRC confirm the enhancing effect of TMZ on susceptibility to ICB [29, 30]. A recent phase II study combining TMZ with Nivolumab for metastatic NENs has shown promising anti-tumor activity [31]. Further validation of this therapeutic strategy is essential through larger clinical investigations.

The MSK-MET cohort highlights that approximately one-third of metastatic pNENs (30.7%) harbor clinically relevant and actionable somatic alterations, expanding potential treatment options. Similarly, WGS of 85 advanced neuroendocrine neoplasms (aNENs) indicates that 49% of aNENs and 55% of advanced PanNETs may offer therapeutic targets based on actionable somatic aberrations within their tumors [10]. Although the link between genomic alterations and specific drugs is conceivable, the impact of such associations on clinical responses remains unclear. While these alterations present promise for novel treatment options in refractory patients, further prospective clinical research is necessary to confirm whether anticancer treatments beyond approved indications, as explored in ongoing studies like DRUP [32], could benefit patients based on the presence of actionable mutations. It’s noteworthy that all four patients with actionable mutations in the PI3K/Akt/mTOR pathway carried *MEN1*/*DAXX* mutations in our cohort. Moreover, tumors with *DAXX* or *ATRX* mutations were significantly correlated with mutations in mTOR regulators in the MSK-MET cohort, consistent with another WGS study of 98 PanNETs [12]. This implies potential differences in drug responsiveness among patients with distinct genomic mutation profiles and underscores a biological link between *MEN1*/*DAXX*/*ATRX* mutations and mutations related to the PI3K/Akt/mTOR pathway during PanNET progression. Importantly, we’ve identified that not all actionable somatic mutations are trunk mutations, highlighting that spatial heterogeneity may lead to a significant underestimation of the detection rate of such mutations from a single-site biopsy, potentially depriving some patients of the opportunity to receive responsive targeted therapy.

Numerous studies have reported the prognostic significance of *DAXX*/*ATRX* somatic mutations in PanNETs [14–16, 33]. Patients with *DAXX*/*ATRX* alterations tend to experience significantly shorter disease-free and recurrence-free survival times, even in cases with small tumors of ≤ 2.0 cm [14]. A meta-analysis [34] examining the impact of altered *ATRX*/*DAXX* genes on prognosis in PanNETs found that, despite an increased propensity towards postoperative relapse in patients with *DAXX*/*ATRX* mutations, no differences were noted in overall survival (OS) compared to *DAXX*/*ATRX* wild-type patients. Interestingly, metastatic patients harboring altered *DAXX*/*ATRX* genes exhibited a trend towards extended OS, consistent with similar results in the MSK-MET cohort [34]. One possible explanation for this variation might be differences in potential drug responsiveness between patients carrying *MEN1*/*DAXX*/*ATRX* mutations and those with wild-type genes. *MEN1*/*DAXX*/*ATRX* mutations are often associated with higher TMB and TNB levels, suggesting a greater potential to benefit from ICB treatment. Tumors with *DAXX*/*ATRX* mutations were significantly correlated with mutations in mTOR regulators, indicating a potential benefit from treatment using PI3K/AKT/mTOR pathway inhibitors, such as Everolimus, an FDA-approved mTOR inhibitor for well-differentiated, advanced PanNETs. However, these trends still require further clinical research verification. Additionally, high TMB/TNB can induce immune activation, making the tumor microenvironment (TME) more active and influencing tumor development and prognosis. Our attempt to decipher inter- or intrapatient differences in the TME using mIHC, while limited by sample size, revealed some noteworthy observations. In patients harboring *MEN1*/*DAXX* mutations, primary tumors exhibited elevated M1 macrophage (CD68 + CD163-) infiltration compared to liver metastases, a distinction not detected in wild-type patients. Single-cell RNA sequencing also revealed spatial heterogeneity in macrophage subtypes between primary and metastatic lesions in PanNETs [35]. These findings suggest potential differences in the molecular mechanisms shaping the TME in PanNETs. Future work should aim to elucidate the characteristics and heterogeneity of the immune microenvironment in PanNETs to support the development of precision immunotherapies for this rare disease.

Two main theoretical models have been proposed to explain tumor metastasis patterns: the linear evolution model and the parallel evolution model [36]. These models are distinguished by two dimensions: (1) the relative timing of the emergence of metastases in the primary tumor (PT); and (2) the expected genetic divergence, characterized by comparing the sum of mutations between the PT and matched metastases. According to the linear evolution model, clones with metastatic capabilities appear late in tumor development and spread when the PT becomes clinically evident, resulting in minimal genetic heterogeneity between the PT and matched metastases. In contrast, the parallel evolution model suggests that metastatic clones or subclones emerge early during tumorigenesis and disseminate at early stages. The primary and metastatic clones evolve in parallel under distinct pressures, leading to noticeable genetic differences between the PT and matched metastases. Previous studies on various cancers, such as breast [7], pancreatic [8], and lung cancers [37], have favored the linear progression model, while colorectal cancers [5] are more consistent with the parallel progression model. However, the trajectory of clonal evolution in PanNET metastasis remains ambiguous. Our study is the first to comprehensively explore this topic in metastatic PanNETs. Our findings revealed that primary-metastatic tumors in PanNETs exhibited significant genome differences, indicating a parallel progression model during metastasis. Regardless of whether it is through a common or distinct origin, the SCIMET model manifests that all metastatic lesions in our cohort, whether lymph nodes or liver metastases, are from early dissemination. This finding is somewhat surprising, given the previously held belief that PanNETs have a favorable prognosis and may even evade adjuvant treatment following radical resection. In general, these findings imply that PanNETs could be intrinsically detrimental, with the potential for invasiveness and metastasis determined at a very early stage. This emphasizes the necessity for targeting the canonical drivers of tumorigenesis. Nonetheless, not every tumor metastasizes, necessitating the urgent identification of biomarkers linked to disease progression and metastasis, which is pivotal for patient risk stratification and making adjuvant treatment decisions.

This study has several limitations. Firstly, the SCIMET model used to estimate dissemination timing assumes a constant positive selection coefficient. Although we selected this coefficient based on earlier research, it is unlikely that the selection pressure in tumors remains consistently stable over time. Future studies should consider variations in selection pressure to assess the significance of incorporating this variation into the model. Secondly, we employed whole-exome sequencing (WES) to elucidate genetic alterations in primary tumors (PT) and matched metastatic samples. However, WES might overlook certain non-coding and structural variants, and whole-genome sequencing (WGS) or an integration of WES with targeted sequencing could yield less biased results. Thirdly, due to the scarcity of PanNETs, acquiring a substantial cohort with matched PT and multiple metastatic samples is challenging. Consequently, this study incorporated a modest patient cohort of only 10 for analysis, especially as the wild-type group only has three cases, and our findings need validation from a larger cohort with multiple metastases. Finally, the potential survival benefit of patients with *MEN1*/*DAXX*/*ATRX* mutations in metastatic PanNETs requires cautious interpretation. This inference was drawn from a univariate analysis of a sizable metastatic PanNET cohort, and the absence of parameters relevant to tumor prognosis—such as differentiation, pathological grade, and metastasis tumor burden—could potentially introduce biases. Thus, this conclusion requires further corroboration.

In summary, we investigated the heterogeneity of the genome and immune microenvironment, as well as the origin and timing of tumor metastasis in metastatic PanNETs. Significant disparities were observed between the *MEN1*/*DAXX* mutant and the wild type concerning genome alterations and clinically actionable somatic mutations. Utilizing the SCIMET model and system evolution analysis, it was shown that most liver metastases are seeded directly from the primary site. Despite differences in the metastatic path between the *MEN1*/*DAXX* mutant and the wild type, it was discerned that PanNETs can be primarily categorized as early disseminated tumors. This suggests the inherent invasiveness and metastatic potential of specific tumors, highlighting the clinical significance of identifying biomarkers related to disease progression and metastasis for tailoring personalized treatment decisions.

### Supplementary Information

**Supplementary Material 1.**

## Data Availability

No datasets were generated or analysed during the current study.

## Abbreviations

PanNETs: Pancreatic neuroendocrine tumors
MRS-WES: Multiregion sampling whole-exome sequencing
NF-PanNETs: Non-functioning PanNETs
PT: Primary tumor
WES: Whole-exome sequencing
mIHC: Multiplex immunohistochemistry
FFPE: Formalin-fixed paraffin-embedded
H&E: Hematoxylin and eosin
ITH: Intratumor heterogeneity
MATH: Mutant-allele tumour heterogeneity
TNB: Tumour neoantigen burden
WGD: Whole-genome duplications
HRD: Homologous recombination deficiency
NMF: Non-negative matrix factorization
NJ: Neighbor-joining
MP: Maximum parsimony
CNV: Copy number variations
MSI: Microsatellite instability
FDR: False discovery rate
SNV: Single-Nucleotide Variant
CCF: Cancer Cell Fractions
dMMR: Mismatch repair deficiency
TLS: Tertiary lymphoid structures
mCRC: Metastatic colorectal cancer
ICB: Immune checkpoint blockade
TMZ: Temozolomide
MSS/pMMR: Microsatellite-stable/proficient mismatch repair
ORR: Objective response rate
DCR: Disease control rate
aNENs: Advanced neuroendocrine neoplasms
OS: Overall survival
