# Correction: Multiregion WES of metastatic pancreatic neuroendocrine tumors revealed heterogeneity in genomic alterations, immune microenvironment and evolutionary patterns

**DOI:** 10.1186/s12964-024-01568-z

**Published:** 2024-03-14

**Authors:** Yu Jiang, Yi-han Dong, Shi-wei Zhao, Dong-yu Liu, Ji-yang Zhang, Xiao-ya Xu, Hao Chen, Hao Chen, Jia-bin Jin

**Affiliations:** 1 Department of Obstetrics and Gynaecology, Shandong Provincial Hospital, Shandong University, Jinan, 250021 Shandong China; 2 Department of Obstetrics and Gynaecology, Shandong Provincial Hospital Affiliated to Shandong First Medical University, Jinan, 250021 Shandong China; 3 The Laboratory of Medical Science and Technology Innovation Center (Institute of Translational Medicine), Shandong First Medical University (Shandong Academy of Medical Sciences) of China, Jinan, 250117 Shandong China; 4 Key Laboratory of Birth Regulation and Control Technology of National Health Commission of China, Shandong Provincial Maternal and Child Health Care Hospital, 328 Jingshi East Road, Jinan, 250025 Shandong China

**Correction: Cell Commun Signal 22, 164 (2024)**

**https://doi.org/10.1186/s12964-024-01545-6**

Following publication of original article [1], the authors identified a production error, whereby Hao Chen (haochendr@126.com) was erroneously omitted from the author group. The author group in this correction article has been updated and the original article [1] has been corrected. The publishers apologize for this error.
