# Supplementary material for: Multiregion WES of metastatic pancreatic neuroendocrine tumors revealed heterogeneity in genomic alterations, immune microenvironment and evolutionary patterns
Source: Cell Commun Signal. 2024 Mar 6;22:164. doi: 10.1186/s12964-024-01545-6 (PMC10916270; doi:10.1186/s12964-024-01545-6)
Supplement: Supplementary file 1 — Supplementary Material 1. [file 12964_2024_1545_MOESM1_ESM.zip › new Figure S.docx]

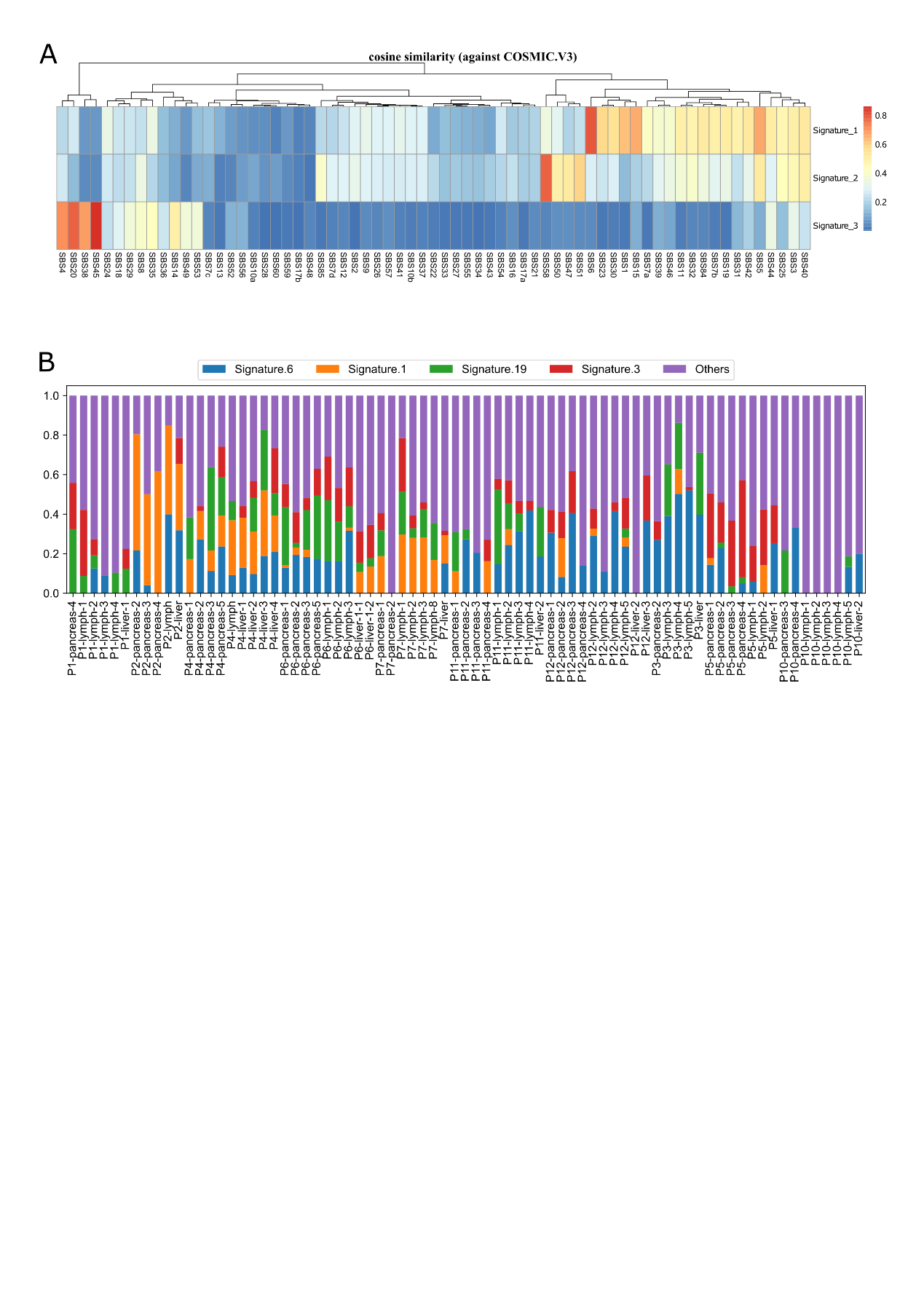


**Supplementary Figure 1.** Mutational signatures

(A) Cosine similarity of de novo mutational signatures against COSMIC Signature V3. The vertical axis represents the de novo signatures, while the horizontal axis depicts the COSMIC V3 signatures. Each tile's color in the figure corresponds to the cosine similarity between the COSMIC V3 signature on the horizontal axis and the de novo signature on the vertical axis. The colors range from blue, indicating the lowest similarity, to cinnabar, representing the highest similarity. Notably, de novo Signature 1 exhibits a strong correlation with COSMIC Signature SBS6 and SBS15 (COSMIC signatures V3), suggesting a defective DNA mismatch repair mechanism.

(B) Relative contribution of the COSMIC single-base substitution mutational signatures (V2). Signatures that are less than 10% are combined into the 'Others' category. Signature 6 (associated with defective DNA mismatch repair) has the highest mean percentage (15.48%).


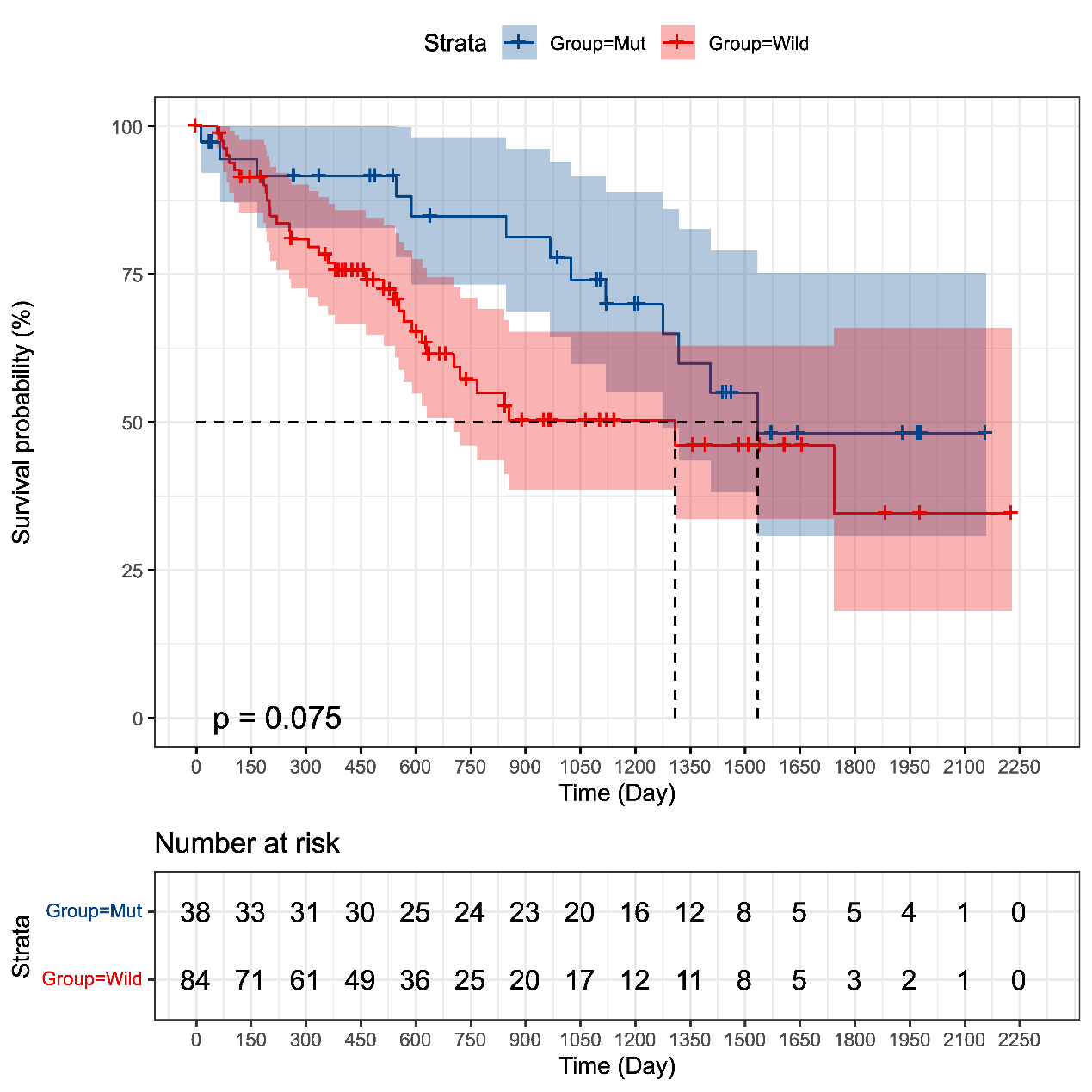


**Figure S2.** The Kaplan-Meier analysis assessed the overall survival of patients in the MSK-MET dataset, distinguishing between those with or without MEN1/DAXX mutations. Patients carrying ATRX mutations were intentionally excluded from this analysis.


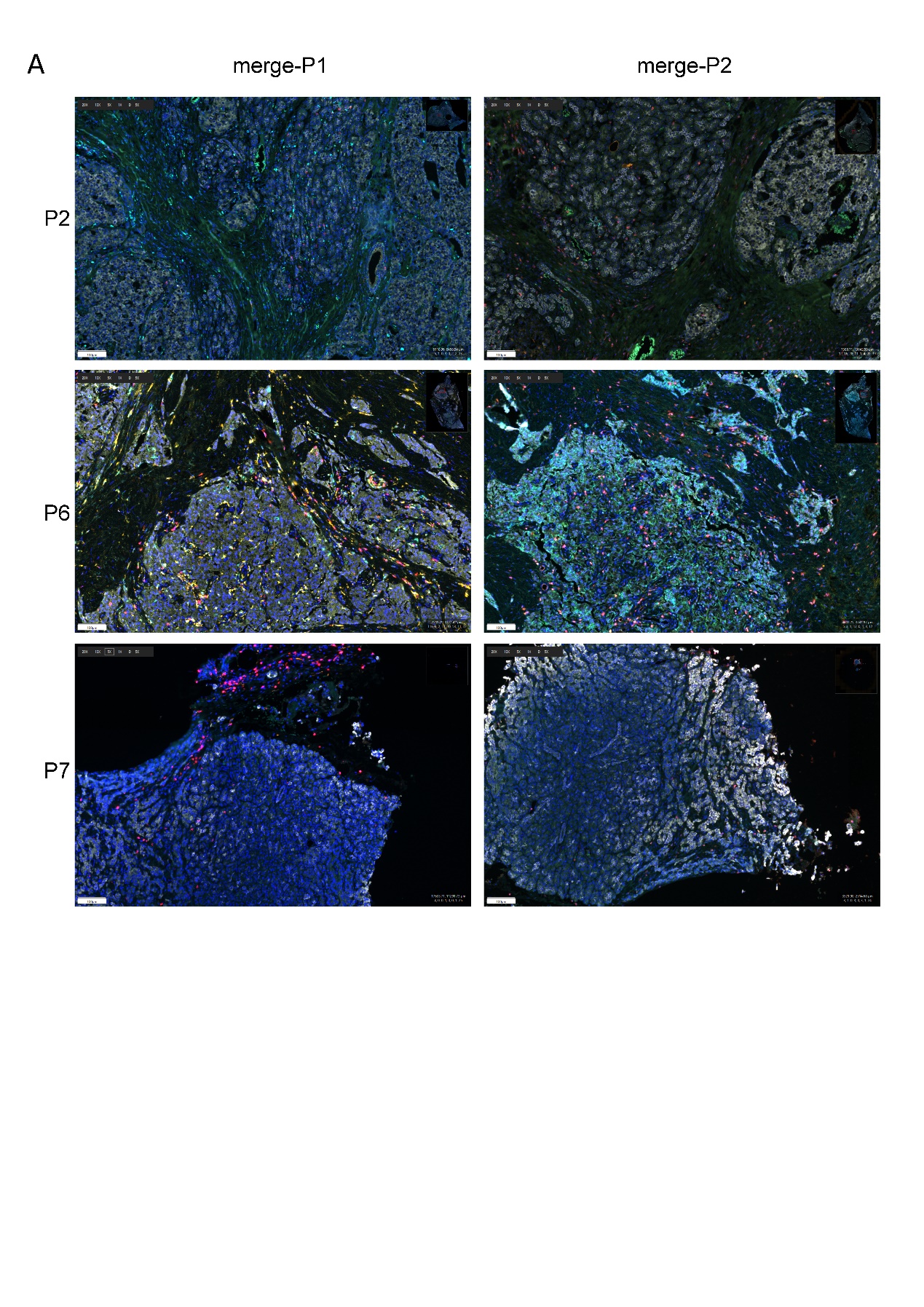


**Figure S3**. Multiplex immunohistochemical (mIHC) staining of primary tumors (P2, P6 and P7) in patients with MEN1/DAXX mutations (PM).

In merge-P1: PD-1, green; PD-L1, yellow; CD8, pink; CD68, cyan; CD163, orange

In merge-P2: CD3, pink; CD4, orange; CD20, green; CD56, cyan; FoxP3, yellow

Blue represents the nucleus


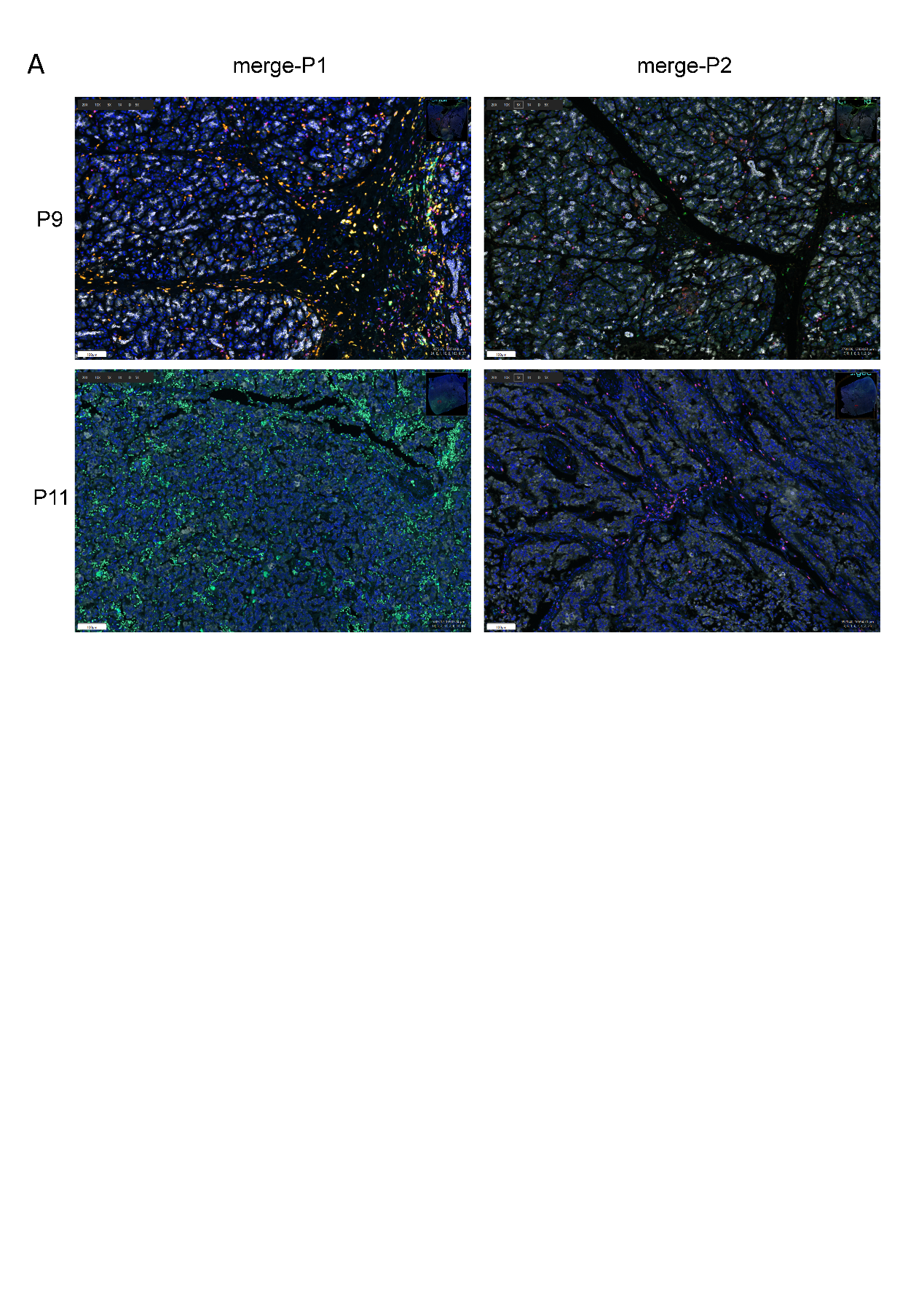


**Figure S4**. Multiplex immunohistochemical (mIHC) staining of primary tumors (P9 and P11) in patients with MEN1/DAXX mutations (PM).

In merge-P1: PD-1, green; PD-L1, yellow; CD8, pink; CD68, cyan; CD163, orange

In merge-P2: CD3, pink; CD4, orange; CD20, green; CD56, cyan; FoxP3, yellow

Blue represents the nucleus


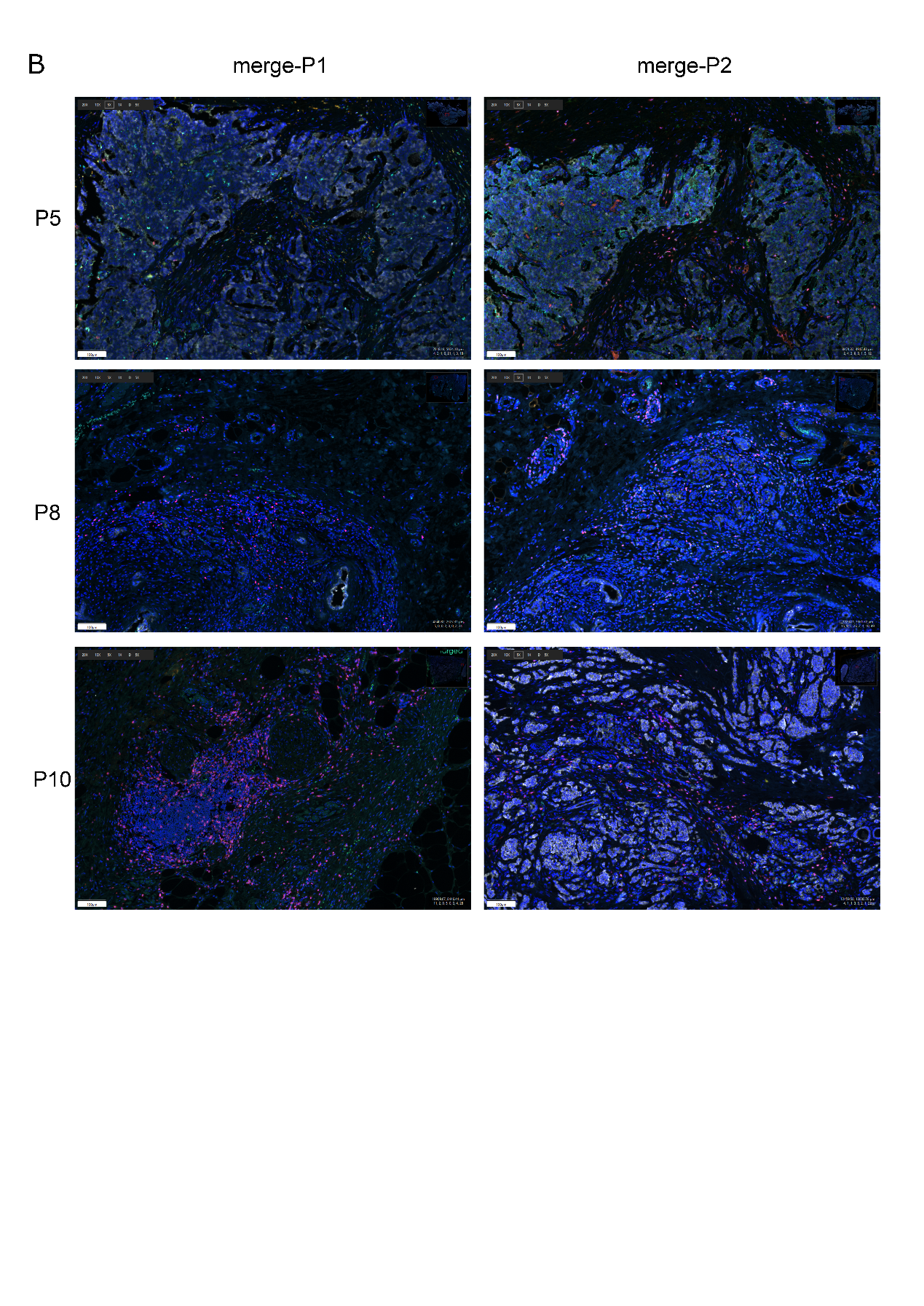


**Figure S5**. Multiplex immunohistochemical (mIHC) staining of primary tumors (P5, P8 and P10) in patients without MEN1/DAXX mutations (PW).

In merge-P1: PD-1, green; PD-L1, yellow; CD8, pink; CD68, cyan; CD163, orange

In merge-P2: CD3, pink; CD4, orange; CD20, green; CD56, cyan; FoxP3, yellow

Blue represents the nucleus


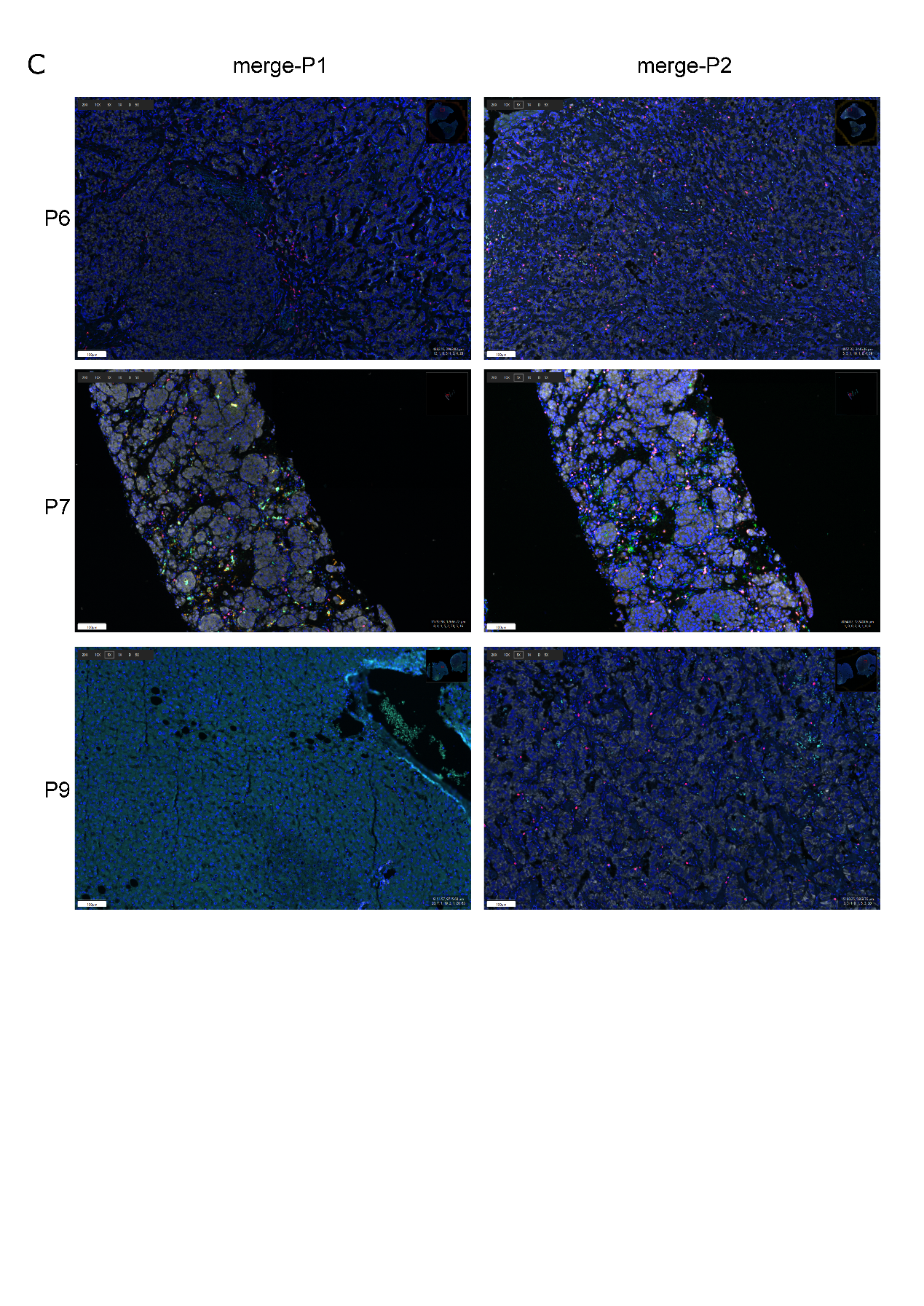


**Figure S6**. Multiplex immunohistochemical (mIHC) staining of liver metastases (P6, P7 and P9) in patients with MEN1/DAXX mutations (MM).

In merge-P1: PD-1, green; PD-L1, yellow; CD8, pink; CD68, cyan; CD163, orange

In merge-P2: CD3, pink; CD4, orange; CD20, green; CD56, cyan; FoxP3, yellow

Blue represents the nucleus


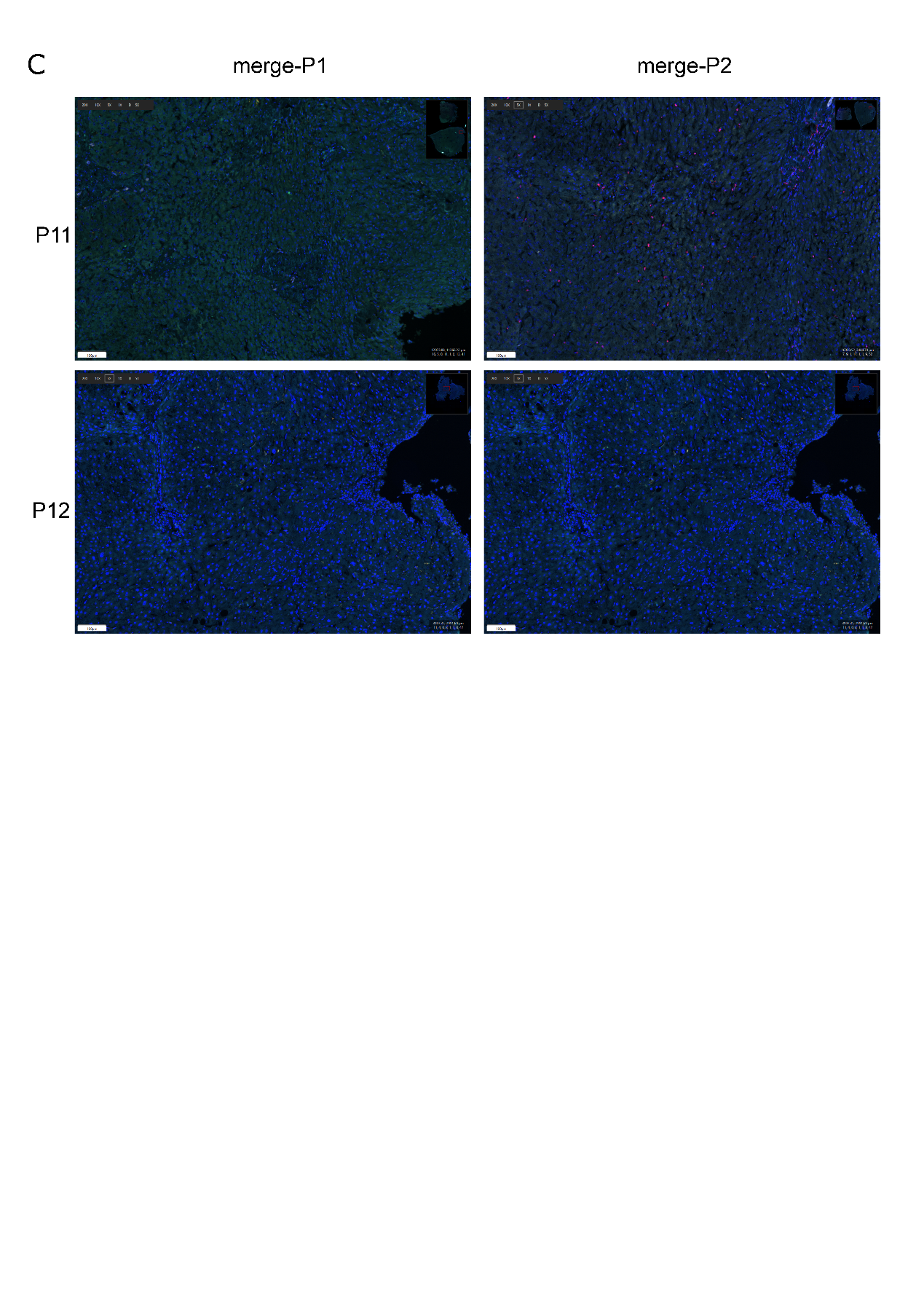


**Figure S7**. Multiplex immunohistochemical (mIHC) staining of liver metastases (P11 and P12) in patients with MEN1/DAXX mutations (MM).

In merge-P1: PD-1, green; PD-L1, yellow; CD8, pink; CD68, cyan; CD163, orange

In merge-P2: CD3, pink; CD4, orange; CD20, green; CD56, cyan; FoxP3, yellow

Blue represents the nucleus


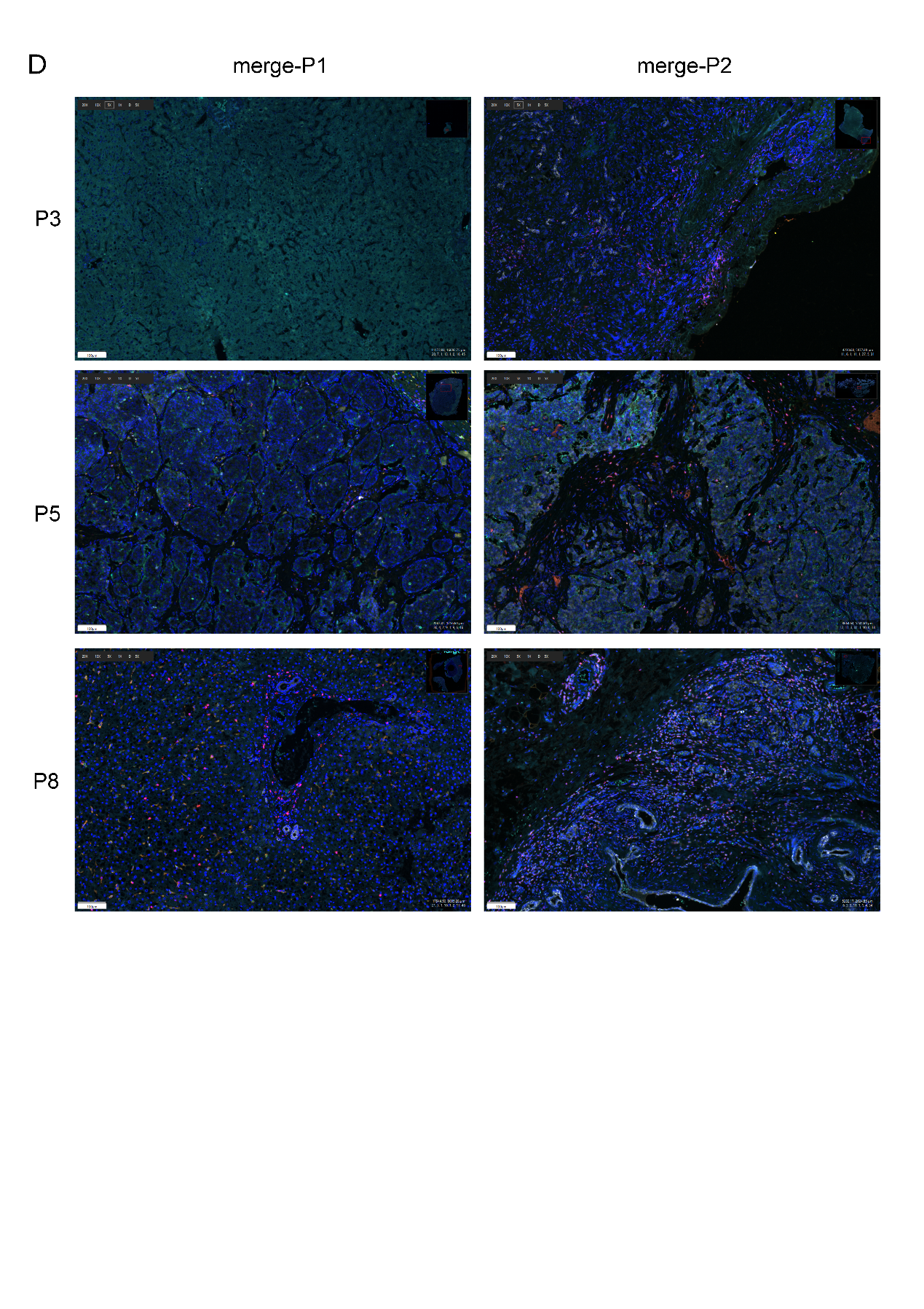


**Figure S8**. Multiplex immunohistochemical (mIHC) staining of liver metastases (P3, P5 and P8) in patients without MEN1/DAXX mutations (MW).

In merge-P1: PD-1, green; PD-L1, yellow; CD8, pink; CD68, cyan; CD163, orange

In merge-P2: CD3, pink; CD4, orange; CD20, green; CD56, cyan; FoxP3, yellow

Blue represents the nucleus


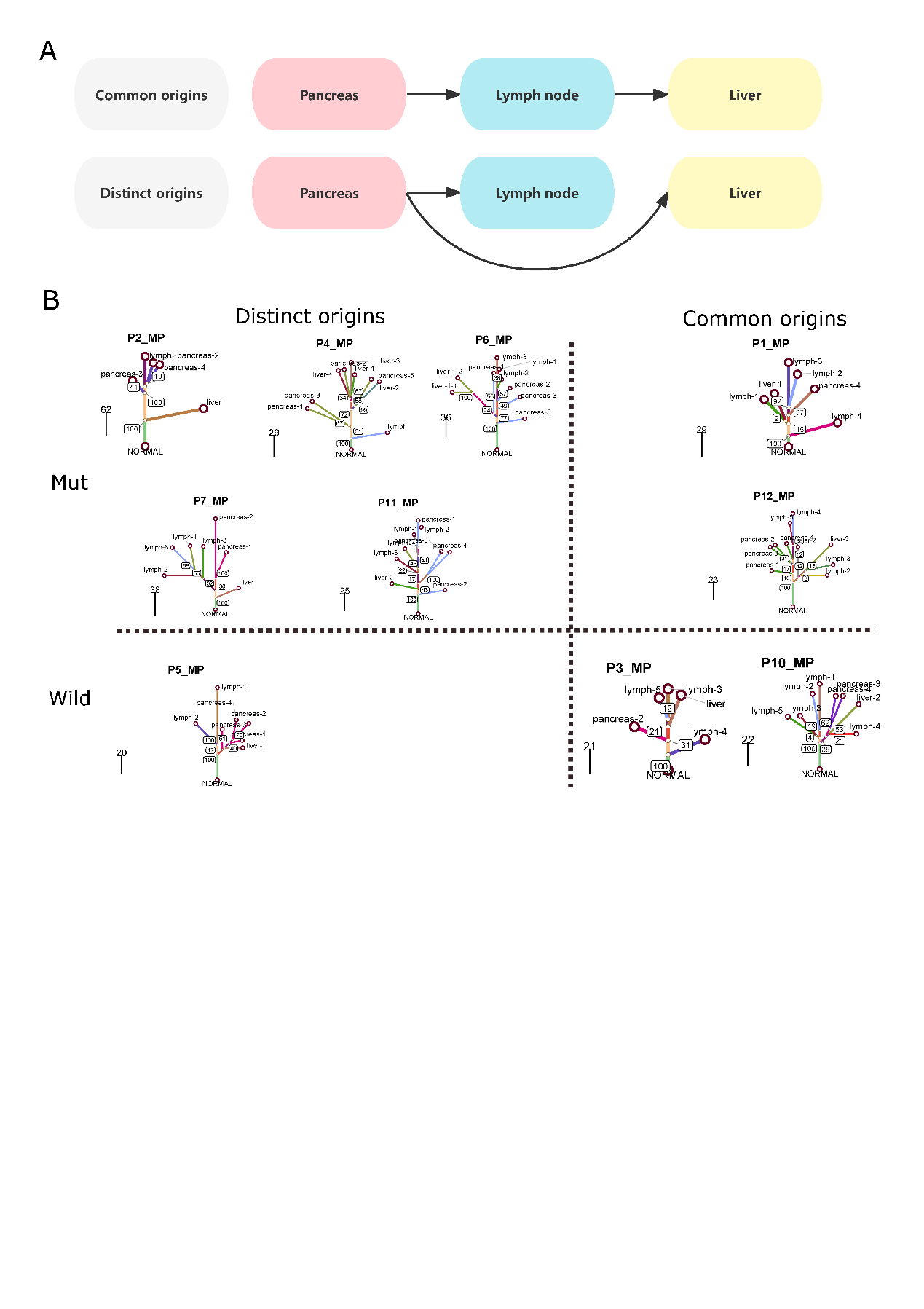


**Figure S9.** Evolutionary origins.

(A) Two tumour migration routes are discernible. In cases of common origins (upper), the phylogenetic tree reveals a clustering of lymph node and distant metastases within the same clade, while primary tumour samples are conspicuously absent. In cases of distinct origins (lower), distant metastasis clusters with the primary tumour sample within one clade, whereas the lymph node metastasis occupies a distinct branch.

(B) The phylogeny was reconstructed using the maximum parsimony method for ten patients. Bootstrapping percentages from 100 repetitions were assigned to each node and are duly labelled.
